# Supplementary material for: Oral Administration of a Select Mixture of Lactobacillus and Bacillus Alleviates Inflammation and Maintains Mucosal Barrier Integrity in the Ileum of Pigs Challenged with Salmonella Infantis
Source: Microorganisms. 2019 May 15;7(5):135. doi: 10.3390/microorganisms7050135 (PMC6560431; doi:10.3390/microorganisms7050135)
Supplement: Supplementary file 1 [file microorganisms-07-00135-s001.pdf]

Table S1. Effects of oral administration of LBB-mix on the incidence of diarrhea in newly weaned pigs before and after *S*. Infantis challenge.

Group[1]/item	Pigs[2]			Pig days				
	At risk	With diarrhea		At risk	With diarrhea		Significance of difference	
	(n)	(n)	(%)	(n)	(n)	(%)	SI	PS
**Pre-challenge**								
CN	8	0	0	54	6	11.11	*	
SI	8	0	0	53	6	11.32		
PS	8	3	37.5	56	12	21.43		
**Post-challenge**								
CN	8	0	0	38	5	13.16	***	***
SI	8	8	100	37	34	91.89		*
PS	8	8	100	40	35	87.5		

[1] CN, control group with sterile physiological saline; SI, oral sterile physiological saline from day 1 to day 7 followed by *S*. Infantis challenge; PS, pretreated with potential probiotic mixture for day 1 to day 7 and followed by *S*. Infantis challenge. [2] In this study, two consecutive fecal scores greater than 4 were identified as diarrhea in the pig. n = 8 pigs per group; Pearson's chi-square test. *$p < 0.05$; ** $p < 0.01$; *** $p < 0.001$.
